# Comparative RNA-sequencing analysis of the prostate in a mouse model of benign prostatic hyperplasia with bladder outlet obstruction

**DOI:** 10.1007/s11010-023-04695-2

**Published:** 2023-03-15

**Authors:** Xiaohu Tang, Zhiyan Liu, Jingwen Ren, Ying Cao, Shujie Xia, Zhaolin Sun, Guangheng Luo

**Affiliations:** 1 Medical College, Guizhou University, Guiyang, 550025 Guizhou China; 2 Department of Urology Surgery, Guizhou Province People’s Hospital, Guiyang, 550002 China; 3 Guizhou Medical University, GuiyangGuizhou, 550025 China; 4 Department of Urology Surgery, Shanghai First People’s Hospital, Shanghai Jiao Tong University, Shanghai, 201620 China

**Keywords:** RNA-seq, BPH, BOO, Testosterone, Estradiol

## Abstract

In ageing men, benign prostatic hyperplasia (BPH) is a chronic disease that leads to progressive lower urinary tract symptoms (LUTS) caused by obstruction of the bladder outlet (BOO). Patients with LUTS (such as increased frequency and urgency of urination) and complications of BOO (such as hydronephrosis and bladder stones) are at risk of serious health problems. BPH causes a rapidly rising burden of LUTS far exceeding that of other urological conditions. Treatment outcomes are unsatisfactory for BPH largely due to the lacking of fully understanding of the pathogenesis. Hormonal imbalances related to androgen and oestrogen can cause BPH, but the exact mechanism is still unknown, even the animal model is not fully understood. Additionally, there are no large-scale data to explain this mechanism. A BPH mouse model was established using mixed slow-release pellets of testosterone (T) and estradiol (E2), and we measured gene expression in mouse prostate tissue using RNA-seq, verified the results using qRT‒PCR, and used bioinformatics methods to analyse the differentially expressed genes (DEGs).

**Supplementary Information:**

The online version contains supplementary material available at 10.1007/s11010-023-04695-2.

## Introduction

Benign prostatic hyperplasia (BPH) is one of the most common diseases in older men, and it affects more than half of all men over the age of 50 years [1]. Its incidence increases further with age, reaching up to 80% in 80-year-old men [2]. Compression of the urethra by an enlarged prostate is the main factor involved in the progression of BPH; this enlargement is characterized by an increase in the number and size of prostate epithelial and stromal cells in the periurethral region [3]. Progressive enlargement of the prostate can lead to compression of the urethra and lead to bladder outlet obstruction (BOO), which can manifest as LUTS [4]. This condition seriously affects the quality of life of patients. BPH is the highest health burden among conditions of the urological system [5]. Although there are many ways to treat BPH, such as alpha1-blockers [6], 5α-reductase inhibitors (5-ARI) [7], and transurethral resection of the prostate (TURP)[8], ejaculation disorders, loss of sexual desire [9], and lower blood pressure [10] can occur with current treatment options [9]; most minimally invasive treatments also destroy the urethra or the prostate, while removing the prostate increases the occurrence of post-operation urinary tract infection [11], affecting sexual function [12] and causing other effects. Additionally, current treatment does not control disease progression in all patients [13, 14].

The pathogenesis of BPH is complex and may be related to the balance in cell proliferation and apoptosis [15], the androgen receptor [16], epithelial mesenchymal transformation [17] and estrogen and androgen imbalance [18], and the molecular mechanisms underlying the clinical phenotype have not yet been fully revealed [19]. Hormonal imbalance related to estrogen and androgen may be one of the important factors causing BPH [20]. As the target organ of sex hormones, androgen and estrogen both regulate the growth of the prostate. It is well known that the prostate is an androgen-dependent organ, but interestingly, testosterone levels reach their peak during early adulthood in men and gradually decrease (1–2% per year)[21], and estrogen levels remain unchanged or slightly decreased, which leads to an increase in the ratio of estrogen to androgen, which can induce the proliferation of stromal cells and their expression of inflammatory factors in individuals with BPH [22]. The estrogen receptor and G protein-coupled estrogen receptor accelerate the progression of BPH by inducing prostatic fibrosis [23]. The coinduction of androgen and estrogen signalling has been shown to accelerate prostatic hyperplasia in dogs and lead to bladder outlet obstruction [24], but the specific mechanism through which estrogen and androgen imbalance affect BPH is still unclear. For a potential breakthrough in BPH treatment, comprehensive research is needed to enable an understanding of the relevant molecular mechanism of BPH development. The existing BPH models rarely have physiological hormone levels associated with the progression of human benign prostatic hyperplasia so it is necessary to establish a mouse model of BPH combined with BOO based on the principle of estrogen and androgen imbalance to further study the pathogenesis of BPH. In addition to 5α-reductase, estrogen and androgen, and their receptor-related genes, BPH is associated with the elevated expression of many genes, which mainly include growth factors; BPH has been found to be associated with abnormal expression of growth factors such as FGF [25], TGF-β [26], EGF [27], and IGF [28]. For example, bFGF can promote the proliferation of prostate fibroblasts cultured in vitro, and prostate stromal cells with high expression of bFGF can stimulate the proliferation of prostate epithelial cells in a paracrine manner [29, 30]. In addition, it was found that the abnormal expression of other genes can promote prostatic hyperplasia; for example, the COX-2/PGE signalling pathway is involved in the progression of BPH [31]. However, the molecular mechanism through which oestrogen and androgen imbalance promotes BPH is not fully understood. RNA-sequencing (RNA-seq) has been widely used in medicine, biology, and other fields [32, 33]; for instance, Magdalena Derbis et al. [34] conducted RNA-seq analyses of the striatum to gain insight into the molecular changes caused by ASO-CCG treatment. Milanez-Almeida et al. [35] recently showed that gene expression data obtained using RNA-seq from The Cancer Genome Atlas (TCGA) could be used to predict survival or the progression-free interval better than classic clinical prognostic factors, such as age at diagnosis, sex, and tumour stage. Therefore, RNA-seq is a key tool for studying disease and biology [36], and it may be beneficial for discovering novel therapeutic targets [37].

Therefore, in this study, a mouse model of BPH combined with BOO was established using testosterone (T) and estradiol (E2) slow-release pellets, and RNA-seq was used to evaluate transcriptomic changes in BPH mice. The reliability of the BPH mouse model was also evaluated by analyses of gross specimens, pathology, and RNA expression, and finally, the key genes, pathways, and infiltrating immune cells were revealed by bioinformatics analysis. In conclusion, the results of this study provide a reference for identifying the potential targets of BPH for treatment.

## Materials and methods

### Environmental conditions and establishment of animal model

**Fig. 1:**
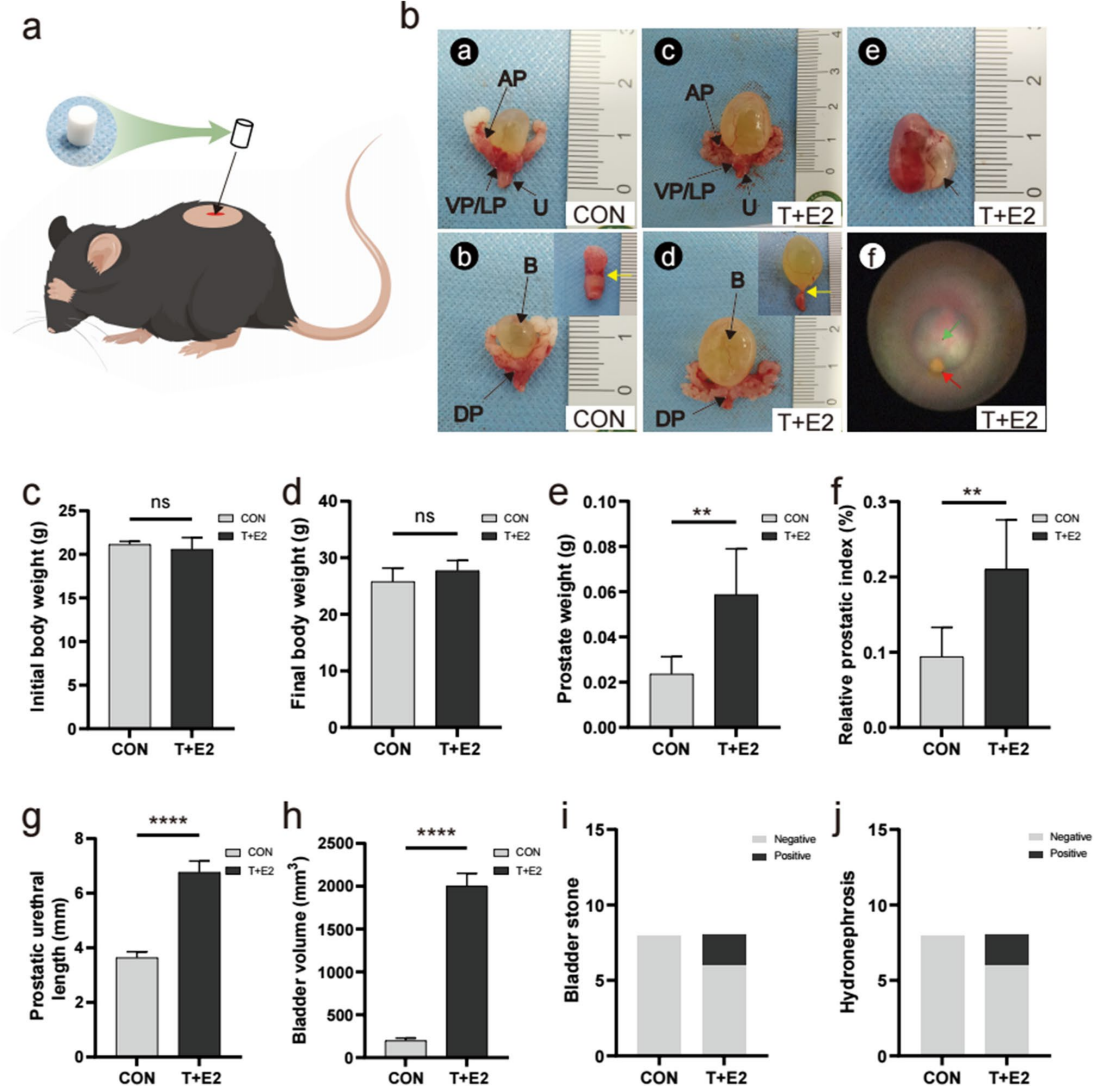
Effects of T and E2 treatment on the gross and urogenital pathology of mice. **A** Mice were implanted subcutaneously with T + E2 slow-release pellets: **B** (1) Ventral view of the prostate and urethra of the CON group; (2) dorsal view of the prostate and bladder of the CON group (the yellow arrowhead indicates the connecting site of the urethra and bladder in the subplot); (3) ventral view of the prostate and urethra of the T + E2 group; (4) dorsal view of the prostate and bladder of the T + E2 group (the yellow arrowhead indicates the connecting site of the urethra and bladder in the subplot); (5) hydronephrosis of the T + E2 group (arrow marks the renal pelvis); (6) endoscopic observation of a stone in the bladder (red arrowhead), and green arrow indicates bladder lumen; **C** initial body weight; **D** final body weight; **E** prostate weight; **F** relative prostatic index: “Prostate weight”/ “Final body weight” × 100%; **G** prostatic urethral length; **H** bladder volume (Formula: volume = Length x width x height x (π/6)); **I** discovery of bladder stones; **J** discovery of hydronephrosis. AP = anterior prostate, VP/LP = ventral prostate/lateral prostate, U = urethra, DP = dorsal prostate, B = bladder. *P < 0.05; **P < 0.01; ***P < 0.001; ****P < 0.0001; ns, not significant

A total of twenty-two 6- to 8-week-old male C57BL/6 mice (Chongqing TengXing Biotechnology Co. Ltd, China) with an average weight of approximately 20 g were used. The animal housing was maintained at 20–25 °C and 40 ~ 75% relative humidity. Indoor lighting consisted of 12 h of light and darkness (split between 8 am and 8 pm), and the animals had free access to food and water. All procedures involving animals were approved by the Ethics Committee of Guizhou Provincial People's Hospital (Ethics approval No. 2022–020). After 1 week of acclimatization, twenty-two mice were randomly divided into two groups (control group: Con group; T + E2 slow-release pellet group: T + E2 group) of eleven mice each; eight mice from each group were used for observation data statistics and prostate pathological examination, and the remaining three mice were used only for RNA-seq. With the aid of a pellet press (Parr Instrument Company, USA), T + E2 was made into a cylindrical solid pill with a diameter of approximately 2 mm and a length of approximately 3 mm, with a total weight of approximately 28.6 mg (T: E2 = 10: 1). After preparing the skin on the back of the mouse, subcutaneous local anaesthesia was performed with 1% lidocaine, and one pill was placed in the incision (Fig. 1A), while the control group received only a dorsal skin incision and suture without the treatment. In the T + E2 group, the pellet in each mouse was replaced with 1 new slow-release pellet each month, and the control group underwent the same operation as described previously.

### Tissue samples

After 12 weeks, the mice were killed with carbon dioxide; the bladder was examined for stones using a cystoscope as we described before [38]; and their urethra, bladder, prostate, kidney, and testis were immediately obtained. The length of the urethra in the prostate was measured using an accurate calliper, and the volume of the bladder when it was full was estimated by measuring the length of the diameter of the bladder and weighing the prostate.

### Haematoxylin–Eosin (HE)

The fixed tissue embedded in paraffin was sectioned into 5-μm-thick sections. After dewaxing and rehydration, sections were stained with haematoxylin and eosin (H&E) according to standard procedures for light microscopic observation. The prostate epithelial thickness, cross-sectional area of the urethral lumen, renal parenchymal thickness, and detrusor muscle thickness of the bladder were measured and analysed by ImageJ software (National Institutes of Health, USA).

### High-throughput RNA-Seq

The total RNA isolated from the prostate tissues (*n* = 3 for each group) was used as input material to prepare RNA samples. Sequencing libraries were generated using the Illumina TruSegTM RNA Sample Preparation Kit (Illumina) following the manufacturer's instructions. The libraries were sequenced on an Illumina HiSeg X-ten System according to the manufacturer's instructions (Shanghai Biotechnology Co.).

### Differentially expressed gene (DEG) analysis

DEG analysis was performed by the R package “DESeq2” for the identification of DEGs using the cut-off criteria of |log2 Fc| > 1 and an adjusted *P* value of < 0.05. Principal component analysis (PCA) plots were generated by the “ggplot2” package in R. PCA and intersample correlation were carried out by the “DESeq2” package in R (version 4.1.3). Correlation heatmap, volcano, and pheatmap plots were plotted in R using the “ggplot2” package and “ComplexHeatmap” package.

### Gene ontology (GO) and kyoto encyclopedia of genes and genomes (KEGG) functional enrichment analysis

The Metascape website (http://metascape.org/gp/index.html#/main/step1) is an effective and efficient portal designed for experimentalists [39]. To elucidate the biological functions of the DEGs, pathway enrichment analysis was performed using KEGG signalling and GO analyses. Metascape was also used to calculate the enrichment terms for up- and downregulated DEGs separately. The top 20 clusters of enriched terms (GO/KEGG terms) are each visualized using a heatmap format, which are coloured based on *P* values.

### Protein‒Protein interaction (PPI) network analysis of DEGs and correlation analyses

Using default parameters, Metascape was used to generate a PPI network with molecular complex detection (MCODE) components, and Cytoscape software (Version 3.9.1) was used for further analysis. We first calculated the top 30 hub genes calculated by each algorithm (betweenness, BottleNeck, degree, radiality, stress) of the cytoHubba plugin in Cytoscape and then screened for genes that were shared with the MCODE seed genes. We continued to explore the relevance of these key genes in normal and hyperplastic prostate tissue (Spearman approach) using the Genotype-Tissue Expression (GTEx) and Gene Expression Omnibus (GEO) databases. GTEx data were obtained from UCSC Xena (https://xenabrowser.net/) and visualized by the package “ggplot2” in R. We downloaded transcriptomic data for all BPH patients from GSE101486, GSE104749, GSE119195, GSE28204, and GSE5377 in GEO. The expression levels obtained from the 5 GEO datasets were normalized using the R package ‘sva’, and the data were visualized using the R package “ggplot2”. Then, we used the genemania plugin in Cytoscape to explore the association between the key genes.

### Gene set enrichment analysis (GSEA) and gene set variation analysis (GSVA)

GSEA is a computational method that determines whether an a priori defined set of genes shows statistically significant, concordant differences between two biological states [40], and the GO/KEGG annotations for GSEA were performed and visualized with the “clusterprofiler” package in R. GSVA, a nonparametric, unsupervised method for estimating gene set enrichment variations in gene expression data, was used to analyse gene set enrichment variations [41]. Then, GO and KEGG results were analysed by the “GSVA” package and visualized by the “ggplot2” R package.

### Generation of the ceRNA network

According to the ceRNA hypothesis, lncRNAs (circRNAs) act as molecular sponges that compete with mRNAs for binding to miRNAs, thus inhibiting their activity [42]. The ceRNA network is widely documented as a posttranscriptional regulatory network that participates in many biological processes. We selected the modules of interest in the PPI network to identify ceRNAs and predicted and visualized the interactions of DEmRNAs-miRNAs and miRNAs-lncRNAs (CircRNA) by StarBase (https://starbase.sysu.edu.cn/) and Cytoscape (Version 3.9.1).

### Assessment of immune infiltration

To explore the immune microenvironment, we performed CIBERSORT analyses to estimate how many infiltrating immune components were present in the samples [43]. We first obtained the mouse immune cell dataset [44], and the “CIBERSORT” package in R software was used to investigate possible associations between the genes and immune cells to predict the relative proportions of 25 infiltrating immune cell subtypes, with a total of 1000 permutations performed.

### Quantitative real-time RT‒PCR

TRIzol (Takara) was used to extract total mRNA, and the PrimerScriptTM RT Reagent Kit (Takara) was used to synthesize cDNA. A SYBR Green PCR kit (Takara Biotechnology Co., Ltd.) was used for qRT‒PCR performed using the CFX96 Touch qRT‒PCR System (Bio-Rad). PCR data were analysed using the 2 − ∆∆Cq method and are expressed as the fold change relative to GAPDH expression levels. All experiments were repeated three times. All primers are listed in Table S1.

### Statistical analysis

GraphPad Prism software (version 9.3.0 for Windows) and R software (version 4.1.3) were used to perform the statistical analyses and data visualization. Wilcoxon tests or Student's unpaired t tests were used to compare data. The correlation analysis was performed by Spearman correlation. Differences were considered significant when *P* < 0.05 (*P < 0.05; **P < 0.01; ***P < 0.001; ****P < 0.0001; ns = not significant, *P* > 0.05).

## Results

### T + E2 treatment induction of BPH and BOO in mice

**Fig. 2:**
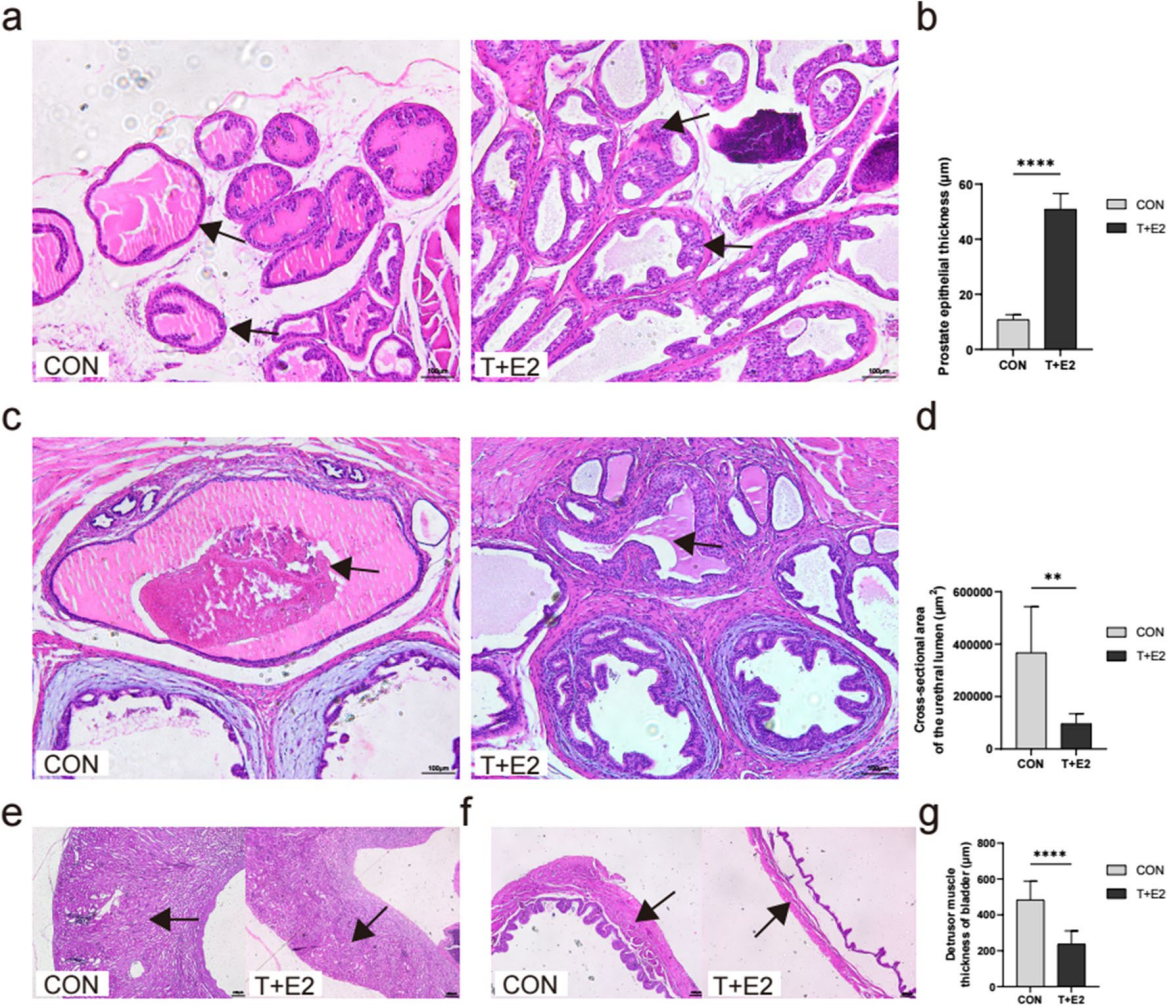
Pathological examination of prostate in mice. **A** Comparisons of HE staining images of prostate tissues between CON and T + E2 group (arrow head: prostate epithelial cells; scale bar = 100 μm); **B** compared with CON group, the thickness of prostate epithelial cells in T + E2 group increased significantly; **C** comparison of the urethral lumen in prostate between the CON and T + E2 groups (arrow head: urethral lumen; scale bar = 100 μm); **D** compared with CON group, the cross-sectional area of the urethral cavity in T + E2 group was significantly reduced; **E** comparison of renal parenchymal thickness between the CON and T + E2 groups (arrow head: renal parenchyma; scale bar = 200 μm); **F** comparison of bladder detrusor between the CON and T + E2 groups (arrow head: bladder detrusor; scale bar = 200 μm); **G** compared with CON group, the detrusor muscle of bladder in T + E2 group was significantly thinner. *P < 0.05; **P < 0.01; ***P < 0.001; ****P < 0.0001; ns, not significant

**Fig. 3:**
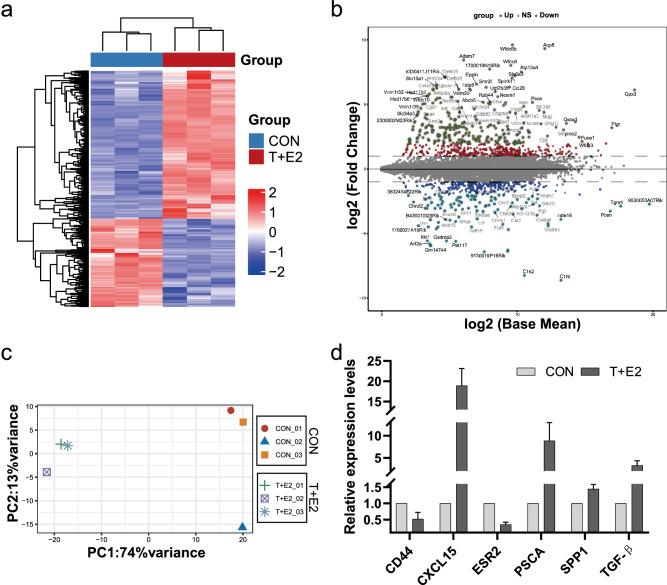
Results of gene expression profile identification and validation of DEGs. **A** Heatmap diagram of DEGs between the CON and T + E2 groups. **B** Volcano plots showing DEGs (the horizontal dotted line marks log2-fold changes of 1 or -1). **C** PCA plot showing the clustering of the samples using all DEGs. **D** Six differentially expressed genes were validated by qRT‒PCR

**Fig. 4:**
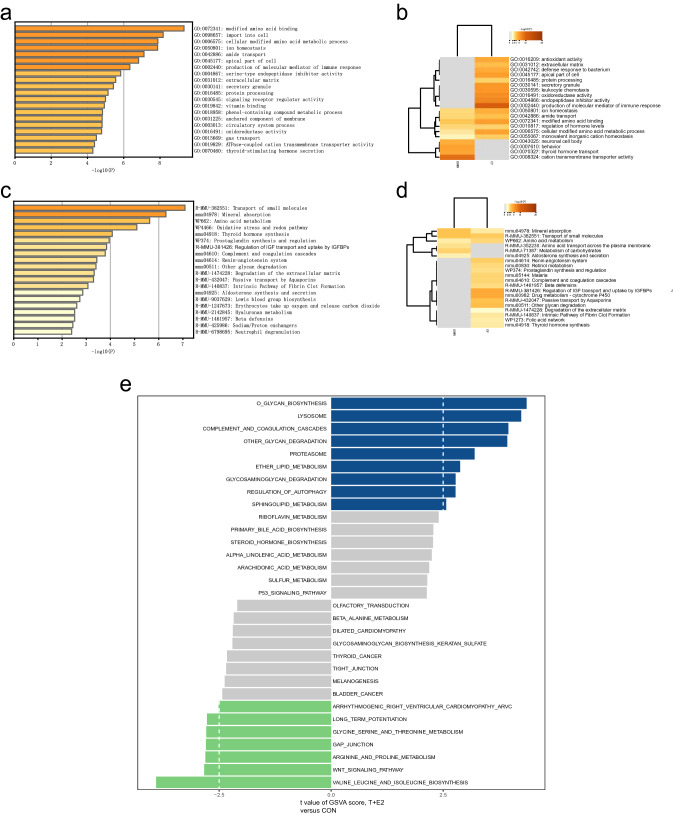
Functional annotation and pathway enrichment analysis. **A** Bar graph of the top 20 enriched significant GO terms (Values represent-log10 p values); **B** GO enrichment analysis of upregulated and downregulated DEGs (Values represent-log10 p values); **C** bar graph shows the top 20 pathways (Values represent-log10 p values); **D** pathway enrichment analysis of upregulated and downregulated DEGs (Values represent-log10 *P* values); **E** bar plot of pathway score calculated by GSVA for the CON group and T + E2 group

**Fig. 5:**
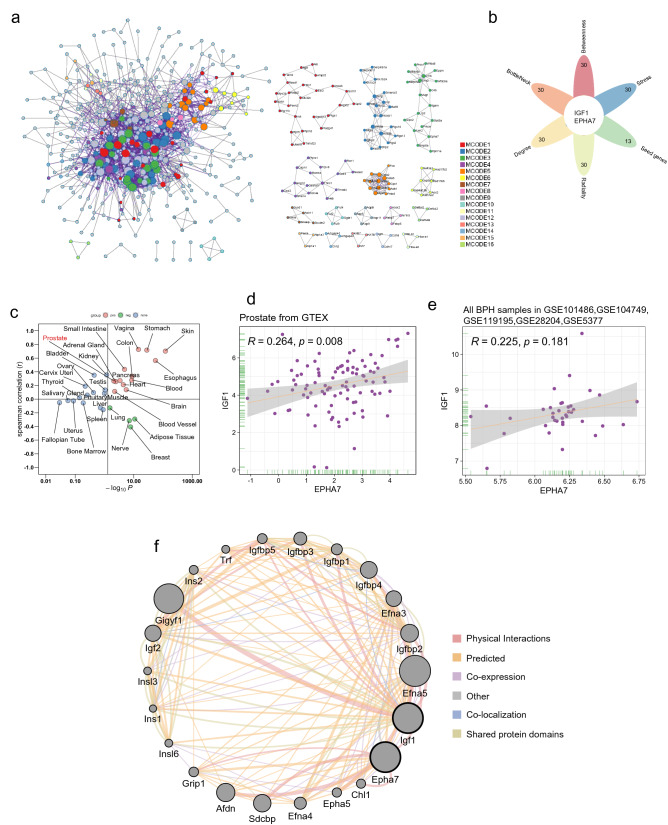
PPI network of the DEGs was generated, and hub genes were analysed. **A** The PPI network and the 16 most significant MCODE components. **B** Flower plot shows the hub genes obtained by 6 algorithms. **C** A comparison of the expression levels of the hub genes in different normal tissues. **D** Scatterplot shows a positive correlation between IGF1 and EPHA7 expression levels in prostate tissue from the GTEx database (*P* = 0.008); **E** Scatterplot shows a positive correlation between IGF1 and EPHA7 expression levels in the prostate of BPH patients from the GEO database (*P* = 0.181); **F** GeneMANIA network: black circles represent inputs into GeneMANIA, and grey circles correspond to GeneMANIA proposed hubs

**Fig. 6:**
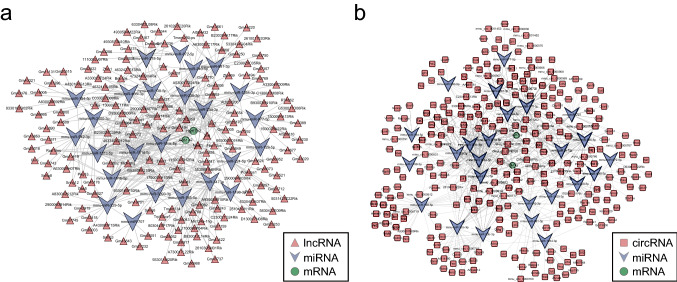
Generation of ceRNA interaction network. **A** lncRNA‒miRNA-mRNA ceRNA regulatory network; **B** circRNA-miRNA‒mRNA ceRNA regulatory network

After 12 weeks of treatment with T + E2 slow-release pellets, all mice survived with good mental status, normal diet and activity, and no hormone-related adverse effects, such as hair loss, but there was a slightly hunched posture during urination in the T + E2 group. The volume of the prostate glands in the T + E2 group was uniformly larger than that in the control group (CON group; Fig. 1B, 2), and bladder volume was significantly higher when the bladder was full (Fig. 1B, 3, 4); the urethra, which was covered by the prostate gland, was narrowed so that more pressure was required to pass the urine from the bladder through the urethra in the T + E2 group (Fig. 1B, 2, 4). Two mice in the T + E2 group suffered from unilateral hydronephrosis (Fig. 1B, 5). More interestingly, we found small stones in the bladder in the T + E2 group, while this phenomenon did not occur in the CON group (Fig. 1B, 6). There were no significant differences in body weights (initial and final body weights) between the CON and T + E2 groups (*P* > 0.05, Fig. 1C–D). The weight of the prostate in the T + E2 group was significantly higher than that in the CON group (+ 148.16%, *P* < 0.01, Fig. 1E), and similarly, the relative prostate index in the T + E2 group also increased (+ 184.22%, *P* < 0.01, Fig. 1F). The urethral length of the prostatic portion in the mice in the T + E2 group was increased (+ 85.90%, *P* < 0.0001, Fig. 1G). The volume of the bladder upon filling was significantly higher in the T + E2 group (+ 905.02%, *P* < 0.0001, Fig. 1H), and two mice with bladder stones and hydronephrosis were found in the T + E2 group (Fig. 1I–J).

### T + E2 treatment causes BPH pathological features

After 12 weeks of treatment with T + E2 slow-release pellets, the epithelial cells of the prostate gland in mice had significantly proliferated, the cytoplasm was full, there were epithelial nodules and an accumulation of epithelial cells, and the lumen of the prostatic ducts was also relatively smaller (Fig. 2A). The epithelial thickness of the prostate gland in the T + E2 group was significantly greater than that in the CON group (+ 369.71%, *P* < 0.0001, Fig. 2B). The urethral opening was relatively narrower in the T + E2 group than in the CON group, with signs of extraluminal compression of the urethral lumen (Fig. 2C) and a reduction in urethral cross-sectional area of approximately 73.83% (*P* < 0.01, Fig. 2D). The parenchymal thickness of the hydronephrotic kidney in the T + E2 group tended to lower than that in the CON group (Fig. 2E). In the T + E2 group, the bladder detrusor was significantly thinner (Fig. 2F) than that in the CON group, with a reduction in thickness of approximately 50.83% (*P* < 0.0001, Fig. 2G).

### DEG analysis and verification

Overall, there were 833 mRNAs with significantly different expression between the T + E2 group and the CON group (Table S2), including 523 upregulated (Table S3) and 310 downregulated mRNAs (Table S4). Hierarchical clustering heatmaps (Fig. 3A) and volcano plots (Fig. 3B) show the differences in gene expression between the two groups. In PCA clustering analysis, samples obtained within the same group were more tightly clustered, and there were significant expression differences between the two groups (Fig. 3C). Samples from the same group were strongly correlated with each other, with significant differences between different groups (Fig. S1). To further validate the sequencing results, the levels of 6 randomly selected mRNAs were measured using qRT‒PCR, and the difference between the two groups was significant (*P* < 0.05) and followed the same trend as the sequencing results, suggesting accurate and reliable sequencing results.

### Functional annotation and pathway enrichment analysis

GO analysis showed that the DEGs were mainly enriched in modified amino acid binding, import into the cell, cellular-modified amino acid metabolic process, ion homeostasis, and amide transport (Fig. 4A, Table S5). Enrichment analysis of the upregulated and downregulated genes revealed that the upregulated DEGs were mainly enriched in the production of molecular mediator of immune response, endopeptidase inhibitor activity, and oxidoreductase activity, while the downregulated DEGs were mainly enriched in cation transmembrane transporter activity, thyroid hormone transport, and neuronal cell body. Up- and downregulated genes were mainly coenriched in ion homeostasis, amide transport, modified amino acid binding, regulation of hormone levels, and cellular modified amino acid metabolic process (Fig. 4B, Table S6).

The results of KEGG enrichment analysis showed that the DEGs were mostly enriched in transport of small molecules, mineral absorption, amino acid metabolism, oxidative stress, redox pathway, prostaglandin synthesis, and regulation (Fig. 4C, Table S7). KEGG enrichment analysis of the up- and downregulated DEGs also revealed that the upregulated DEGs were mainly enriched in the regulation of insulin-like growth factor (IGF) transport and uptake by insulin-like growth factor binding proteins (IGFBPs), passive transport by aquaporins, and other glycan degradation, while the downregulated DEGs were mainly enriched in amino acid transport across the plasma membrane, metabolism of carbohydrates, and aldosterone synthesis and secretion. The up- and downregulated DEGs were mainly coenriched in mineral absorption, transport of small molecules, and amino acid metabolism (Fig. 4D, Table S8).

GSEA was performed for GO and KEGG analysis, and the results showed that the main terms were regulation of endopeptidases and peptidases and the complement and coagulation cascade pathways (Fig. S2–S3, Tables S9–10).

GSVA scores for GO items were high for positive regulation of cholesterol esterification (biological process (BP)), cellular response to insulin-like growth factor stimulus (BP), endoplasmic reticulum tubular network membrane (cellular component (CC)), phospholipid translocating ATPase complex (CC), and phosphate ion transmembrane transporter activity (molecular function (MF)) in the T + E2 group. In the CON group, mitochondrial translational elongation (BP), positive regulation of 3’UTR-mediated mRNA stabilization (BP), elastic fibre (CC), cell body fibre (CC), and leucine transmembrane transporter activity (MF) were scored high via GSVA (Fig. S4-A, B, C, Tables S11–13).

GSVA suggested that o-glycan biosynthesis, lysosome, complement and coagulation cascades, proteasome, and regulation of autophagy were scored high in the T + E2 group. In the CON group, valine, leucine, and isoleucine biosynthesis; Wnt signalling pathway; arginine and proline metabolism; gap junctions; and glycine serine and threonine metabolism were highly scored (Fig. 4E, Table S14).

### Construction of the PPI network and analysis of hub genes

The PPI networks constructed using Metascape, with 479 nodes and 1607 edges, were also divided into 16 significant modules based on different GO clusters (Fig. 5A, Table S15) and the genes in each module (Table S16). Module 5 was the most central module associated with antioxidant activity. Previous studies have demonstrated a close relationship between antioxidant activity and BPH [45]. All 13 genes from module 5 had the highest Macode score of the top 13, and the genes from module 5 were also used to generate the subsequent ceRNA network. Next, we used the five algorithms in Cytoscape’s plugin “Cytohubba” to identify the top 30 genes for each and the key genes IGF1 and EPHA7 that were shared with the seed genes of the 16 important modules (Fig. 5B). In normal tissues obtained from the GTEx dataset, the levels of these 2 genes showed different correlations in different tissues (Fig. 5C), with a significant positive correlation between the levels of these 2 genes in prostate tissue (*P* < 0.05, Fig. 5D). We further examined the transcriptional data obtained from all BPH samples from 5 datasets (37 samples in total) from GEO and found a positive but nonsignificant correlation between the levels of 2 genes (*P* > 0.05, Fig. 5E, Table S17). A network of Igf1 and Epha7 interactions was generated using the genemania plugin in Cytoscape, and the 2 genes were found to be linked in various ways, including physical interactions, prediction, and coexpression.

### Construction of the ceRNA network

To explore the molecular regulatory mechanisms of DEmRNAs within the module of interest, lncRNA‒miRNA-mRNA and circRNA-miRNA‒mRNA regulatory networks were established. lncRNA-related ceRNA networks were generated, involving 199 molecules and 445 interactions, including those of 2 DEmRNAs, 172 predicted lncRNAs, and 25 predicted miRNAs (Fig. 6A, Tables S18–19). The other circRNA-associated ceRNA network, involving 300 genes and 608 interactions, included 2 DEmRNAs, 273 predicted circRNAs, and 25 predicted miRNAs (Fig. 6B, Tables S18, 20). ceRNA networks can enable an understanding of the regulatory relationships of these key genes and help discover more potential biological targets. For example, we focused on Hsd17b2, which could be regulated by miR-181a-5p, while lncRNA NEAT1 and circRNA Snx5 can regulate Hsd17b2 by competing for miRNA binding sites.

### Immune cell infiltration

**Fig. 7:**
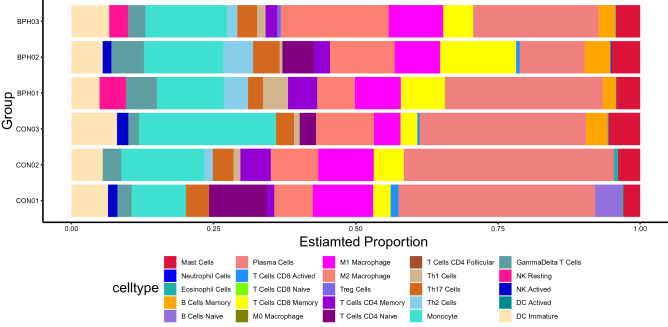
Proportion of 25 immune cells in each sample from the CON and T + E2 groups

Using the CIBERSORT algorithm, we compared the infiltration levels of 25 subsets of immune cells in the prostate tissues between the two groups and found that all samples were infiltrated by multiple types of immune cells; for example, plasma cells and monocytes were the predominant immune cells in all samples, while eosinophils were less represented (Fig. 7), but there was no significant difference in the proportion of individual types of infiltrating immune cells when the two groups were compared (*P* > 0.05, Fig. S5).

## Discussion

During adulthood, the prostate is the only solid organ that continues to grow continuously [46]. BPH has been found to be associated with quality-of-life (QoL) alterations and health problems in elderly men [47, 48]. In our study, mice treated with T + E2 showed not only the gross and pathological features of BPH but also features that have only observed in patients with progressive BPH, such as elongation and narrowing of the urethra in the prostate area, as well as bladder stones, which is consistent with secondary stones, and hydronephrosis and bladder wall thinning caused by BOO [49–53]. The elongation and narrowing of the urethra in the prostate might be a key factor in the development of BOO in BPH mice, and the elongation of the urethra at the junction with the bladder was particularly pronounced and might be related to the lack of fixation of the urethra in this area by tissues, such as smooth muscle, and the pull of the dilated bladder. The T + E2-induced mouse model of BPH has greater advantages than the rat model, as the characteristic alterations associated with BPH combined with BOO more closely resembled those in humans, while similar findings have not been reported for the steroid hormone-induced BPH rat model. In addition, the genetics of the mice was more fully explored, and the mature gene editing system in mice is more conducive to assessing the impact of changes in genetic information on biological functions [54, 55]. The protocol for inducing BPH mice with BOO in this study had the shortest known induction time, requiring only three subcutaneous implantations of slow-release pellets with a total induction time of 12 weeks, which leads to a lower risk of infection than traditional daily injections of sex hormones, facilitating the mass production of BPH-combined BOO mice. The T + E2-induced mouse model of BPH with BOO can provide a reliable research tool for the study of mechanisms related to BPH.

A proportion of patients with BPH who are treated clinically with medication eventually require surgery, accounting for approximately 10% [56], which reflects the limitations of current pharmacological treatment options. The imbalance of androgen and oestrogen was considered to be one of the important pathogenic mechanisms underlying BPH, but there was no comprehensive and effective treatment for the imbalance [57], and the search for new therapeutic targets had become an urgent matter, but there was a lack of large-scale transcriptomic screening. This type of screening is indispensable for an in-depth study of the molecular mechanisms involved in androgen- and oestrogen-induced BPH. By RNA-seq, 833 significantly differentially expressed genes were identified between the two groups. In the results of GO enrichment analysis, we found that upregulated DEGs were significantly enriched in redox processes, which was consistent with previous studies that the redox system was associated with the development of BPH [58]. The destruction of redox balance is easy to induce oxidative stress [59]. Oxidative stress is also considered to be one of the mechanisms that cause early pathological changes during prostate hyperplasia [60]. Oxidative stress can promote prostate hyperplasia by affecting the apoptosis pathway of prostate cells [61], inducing prostatitis [62, 63], and accelerating prostate proliferation [64] by activating the PI3K/Akt signalling pathway. Androgen can induce oxidative stress by increasing free radicals [65] and physiological amount of estrogen has antioxidant effect, but over-physiological amount of estrogen may induce oxidative stress by causing mitochondrial dysfunction [66]. However, the relationship between the ratio of estrogen/androgens and oxidative stress is still unknown, and further study is needed. Antioxidant stress may become a new therapeutic target for BPH; for example, previous studies have found that the lipid extract from the fruit of the Royal Palm of Cuba may treat prostatic hyperplasia through antioxidation [67]. GO terms enriched in the downregulated DEGs were associated with ion transport [68], whereas the upregulated DEGs were enriched in hormonal regulation, and previous studies had demonstrated the involvement of both androgens and oestrogens in the progression of BPH[23, 69]. In addition, the results of KEGG analysis suggested that the most significant pathway was small molecules, which had been shown in previous studies to influence the cell cycle, apoptosis and proliferation [70–72], whereas the upregulated DEGs were most significantly enriched in pathways associated with IGFs and IGFBPs, which have been previously shown to be associated with BPH [28, 73], and inhibition of IGF-1 secretion inhibited the proliferation of prostate epithelial cells [29]. Up- and downregulated DEGs were also predominantly coenriched in small molecule compound-related pathways, suggesting that this pathway might play an important role in steroid hormone-induced BPH. It is well accepted that BPH is an androgen-dependent condition, as castrated individuals do not develop BPH [74]. In the prostate, 5 α-reductase converts testosterone into dihydrotestosterone, which in turn combines with the androgen receptor to promote prostatic hyperplasia. Steroid signalling through the androgen receptor is considered to be the key regulator of prostatic hyperplasia [75, 76]. In addition, oestrogen signalling plays an important role in the pathophysiology of prostatic hyperplasia, and oestrogen can regulate the proliferation of primary stromal cells and their expression of inflammatory factors during BPH [22, 31]. By increasing oestrogen to androgen ratios, upregulated AR expression may increase the sensitivity of the prostate to androgens, resulting in prostate hyperplasia [31]. To further explore steroid hormone-induced genomic differences in mice, we used GSEA and GSVA. GSEA results showed that terms enriched for GO and KEGG were mainly associated with the regulation of endopeptidases and peptidases and complement and coagulation. GSVA showed that enriched GO and KEGG terms were mainly associated with cellular responses to insulin-like growth factor stimulation and autophagy, which is consistent with previous studies that have shown that BPH is associated with insulin-like growth factor-mediated hormone imbalance [77] and impaired autophagy [78].

The PPI network generated in this study identified 16 important modules; for example, module 6 was mainly associated with oxidoreductase activity and the steroid metabolic process, and previous studies have shown that dysregulation of redox homeostasis was associated with the pathogenesis of prostate hyperplasia [58]. It is known that the development of BPH is dependent on steroid hormones [79]. The key genes IGF1 and EPHA7, important seed genes in the modules and hub genes in several algorithms, were also identified, and previous studies have shown that IGF1 is associated with the development of BPH [64, 80, 81]. Findings involving EPHA7 have not been reported in most studies of BPH, although one study found that EPHA7 expression was upregulated in BPH tissue or normal tissue compared to most PCa samples [82]. In correlation studies, we found different correlations between IGF1 and EPHA7 levels in different tissues, implying that they may have different interregulatory effects; the levels of these genes were positively correlated in organs of the digestive system, such as the small intestine, stomach, colon, pancreas, and oesophagus. However, they were negatively correlated in adipose tissue. In prostate tissue, IGF1 levels were positively correlated with EPHA7 levels. Then, we studied all BPH samples from 5 GEO datasets and found that the levels of the two genes were also positively correlated, but this result was not statistically significant, indicating the need for further validation with more samples. Further study of the interrelationship of the 2 genes revealed a high degree of interrelationship in a biological network with the 2 genes as the core; this interrelationship involved physical interactions, predicted, coexpression, colocalization, and shared protein domains in the network. We found many members of the Eph family and IGFBP family. As the largest family of tyrosine kinases, the Eph receptor has 14 members, and the binding site of its ligand (Ephrin) is located on the cell membrane [83]. In addition to modulating IGF bioactivity, IGFBP family members have independent biological actions [84], and it was predicted by the network that IGF-1 might interact directly or indirectly with multiple IGFBP family and Eph family members. The network predicted that IGF-1 might interact directly or indirectly with several IGFBP and Eph family proteins, suggesting that these interactions might be related to the regulation of cell-to-cell crosstalk.

Based on the ceRNA hypothesis, lncRNAs (circRNAs) might act as ceRNAs by acting as sponges for miRNAs and thus indirectly regulate mRNA expression. The expression of mRNAs in this network is regulated directly and indirectly by miRNAs and lncRNAs (circRNAs)[85]. Many noncoding RNAs in the generated ceRNA network might be involved in regulating these key genes, which in turn can influence the regulation of steroid hormone-induced BPH. For example, in the lncRNA-related ceRNA network, NEAT1 can regulate the expression of Hsd17b2, an mRNA related to steroid metabolism, via miR-181a-5p [86]. Steroid hormones were inactivated by HSD17B2, and their balance was regulated in a variety of tissues by this molecule [87]. The elevation in HSD17B2 levels may have been due to the supraphysiological dose of steroid hormone used in this experiment. In the circRNA-related reRNA network, circRNA Snx5 regulates Aldh1a2 (a retinoic acid synthase) via miR-129-5p, and elevated Aldh1a2 promotes retinoic acid synthesis, while retinoic acid (a vitamin A metabolite) also exhibits anti-inflammatory effects by preventing oxidative stress [87]; these findings suggest feedback regulation by mouse prostate tissue in response to intense oxidative stress. The above findings also suggest that steroid hormone-induced BPH in mice was regulated by a dynamic balance of oxidative stress and antioxidative stress.

In the immune infiltration analysis, we found no significant difference in the proportion of 25 immune cells after T + E2 treatment and similar levels of immune infiltration in both groups. Erin M. McAuley et al.[88] used T + E2 to treat mice and similarly found no effect on the distribution of immune cells in the prostate. Human BPH tissue contains infiltrating T lymphocytes, B lymphocytes and macrophages, which might drive fibromuscular growth during BPH by activating and coordinating the release of cell factors [89]. The time of our prostate sample collection might not have coincided with the time of immune infiltration, and more samples are needed to confirm the findings regarding immune infiltration in the model. The shift from experimental animals to clinical application needs to be continuously explored and validated.

## Conclusion

In conclusion, we established a mouse model of BPH combined with BOO that is simple to manipulate and suitable for mass production. The model is associated with the specific alterations in histology and general pathology associated with BPH and provides a research platform for further studies of BPH. In this study, we also identified important genes and pathways involved in steroid-induced BPH and explored intermolecular regulatory relationships and immune features, and these results might provide new insights into the search for therapeutic targets for BPH.

### Supplementary Information

Below is the link to the electronic supplementary material.

Supplementary file1 (RAR 4598 KB)

## Data Availability

Raw data files have been deposited to the Gene Expression Omnibus Database (www.ncbi.nlm.nih.gov/geo/), and accession number is GSE218403.
